# A modified Delphi exercise in physician-perceived risk factors for drug-induced pneumotoxicity in patients with rheumatological disease

**DOI:** 10.1186/s12890-024-03287-0

**Published:** 2024-10-31

**Authors:** Manjit K. Cartlidge, Kevin K. Brown, Nazia Chaudhuri, Tamera J. Corte, Phillipe Dieudé, Levin John, Clive Kelly, Dinesh Khanna, Euan McRorie, Lisa Nicol, Gareth Stewart, Simon L. F. Walsh, Marlies Wijsenbeek, Nik Hirani, PASSWRD participants, George W Chalmers, Janardhana Golla, Charlotte Hyldgaard, Benjamin Chaigne, Patricia López Miguel, Elisabeth Bendstrup, Roberto G Carbone, Albert Selva-O’Callaghan, Nazia Chaudhury, Enrico Selvi, Tonya Russell, Pedro Ferreira, Suranjan Mukherjee, Carrie Kah-Lai Leong, Tiago Alfaro, Patricia E Carreira, Devesh J Dhasmana, Paolo Cameli, Wim A Wuyts, David Bennett, Luca Novelli, Divya C Patel, Ahmed Fahim, Margaret L Wilsher, Adrian Shifren, Maria L. Padilla, Carolina Muller, Sergey Avdeev, Marta Dzhus, Ilias C Papanikolaou, Yoshinori Tanino, Harvard Fretheim, Alexandra Balbir-Gurman, Vanesa Vicens-Zygmunt, Mark G Jones, Michael Perch, Daniel Brito de Araujo, Edoardo Conticini, V Keshavan, Shinyu Izumi, Meena Kalluri, Amy Hajari Case, Alice M Turner, Marko Baresic, Gouri M Koduri, Alexandre Franco Amaral, Glenn Eiger, Mauricio Salinas, Mario Sergio Nunes, Gin Tsen Chai, Simone Scarlata, Elżbieta Radzikowska, Toby M Maher, Maurizio Benucci, Katherine J Myall, Jesper Rømhild Davidsen, David Launay, Dr Emma L Culver, Horacio Matias Castro, HJ Gayathri Devi, Caterina Naclerio, Ulrich A. Walker, Felix Chua, Estrella Garcia Gonzalez, Sandra Fabiana Montoya, Sara Madelaine Carty, Eoin P Judge, Sarah L O’Beirne, Kerri A Johannson, Philippe Camus, Semra Bilaceroglu, Philip V Gardiner, Lisa M Nicol, Álvaro Garcia Martos, Diego Castillo, Randolph J Lipchik, Fotio Drakopanagiotakis, Jens Vikse, Maria Teresa Rio Ramirez, Danielle Antin-Ozerkis, Rebecca Grainger, Gareth A Stewart, Raphael Borie, Aditya Agrawal, Angela Ceribelli, Alfredo Guillen, Shigeki Saiton, Keisuke Tomii, Tracy Luckhardt, Kristin B Highland, Ana Maria Gheorghiu, Martin Kolb, Claudia Cobilinschi, Richard Mathew Jones, Sergio Campainha, Edoardo Rosato, Rosario Foti, Pierre-Antoine Juge, Shital Patil, Nasser Al Busaid, Simona Rednic, Liudmila Garzanova, Joshua J Solomon, Ali Fuat Kalyoncu, Alessandra Della Ross, Dijana Perkovic, Yasemin Kabasakal, Nesrin Mogulkoc, Su-Ying Low, Godoy, Lisa G Spencer, Alain Delobbe, Claudia Lucia Toma, Elvis Hysa, Davide Mohammed Reza Beiga, Yuko Waseda, Venero MdC, Helen Parfrey, Emma Derrett-Smith, Silvia Grazzini, Christopher J Ryerson, Michele Iudici, E J Nossent, Corrado Campochiaro, Abdulla Al-farttoosi, Andreina Manfredi, Alejandro Robles-Perez, Ivo van der Lee, Nik Hirani, Alberto Sulli, Kristina Frketic Marovic, Peter Saunders, Vera Bernardino, Toshiaki Matsuda, Pilar Rivera-Ortega, Virginia Berlengiero, Jadranka Morovic-Vergles, Esen Kiyan, Elisabetta Balestro, Armando Gabrielli, Marco Sebastiani, Paola Confalonieri, Bruno Crestani, HC Blum, Gunnar Gudmundsson, Anjali Crawshaw, Alejandro Robles-Perez, Simon M Stebbings, Sameep Sehga, Deborah Assaya, Hilario Nunes

**Affiliations:** 1 Edinburgh Lung Fibrosis Clinic, Royal Infirmary of Edinburgh, Edinburgh, UK; 2 Department of Medicine, National Jewish Health, Denver, CO USA; 3 Department of Health and Life Sciences, University of Ulster, Derry-Londonderry, UK; 4 Royal Prince Alfred Hospitaland, University of Sydney, Camperdown, Australia; 5 Bichat Claude-Bernard Hospital, APHP University Paris Cite, Paris, France; 6 Centre for Integrative Omics Data Science (CIODS), Yenepoya University, Mangaluru, India; 7 James Cook University Hospital, Middlesbrough, UK; 8 University of Michigan, Ann Arbor, USA; 9 Western General Hospital, Edinburgh, UK; 10 Institute for Regeneration and Repair, Centre for Inflammation Research, University of Edinburgh, Edinburgh, UK; 11 National Heart and Lung Institute, Imperial College, London, UK; 12 Erasmus MC Centre of Expertise for Interstitial Lung Diseases and Sarcoidosis, Erasmus University Medical Centre, Rotterdam, The Netherlands

**Keywords:** Drug-induced pneumotoxicity, Drug-induced interstitial lung disease, Interstitial lung disease, Rheumatological drugs, Pneumotoxicity

## Abstract

**Background:**

Drugs used to treat rheumatic disease are associated with pneumotoxicity (drug-induced lung disease), but little is known about associated risk factors.

**Aim:**

To determine expert physician-perceived risk factors for developing pneumotoxicity in patients with rheumatologic conditions.

**Methods:**

A modified international 3-tier Delphi exercise was performed. Tier 1 determined patient and drug variables that physicians perceive to be risk factors. Tier 2 determined degree of risk associated with the Tier-1 derived variables. Tier 3 aimed to internally validate and stratify exemplar cases into risk categories.

**Results:**

134 pulmonologists and 49 rheumatologists responded to Tier 1;157 physicians completed all tiers. Perceived risk factors included: drug type; history of previous pneumotoxicity; age; smoking; underlying rheumatic disease type and activity; renal function; pulmonary hypertension; left ventricular failure;presence, nature, severity and progression of pre-existing interstitial lung disease. Tier 2 data stratified these variables into risk profiles e.g. never versus current smoking was perceived as low and high risk respectively. An example of perceived high risk resulting from Tier 3 is a 75-year-old current smoker with high-activity rheumatoid arthritis (RA) with severe, progressive ILD being started on methotrexate. A perceived low risk is a 75-year-old currentsmoker with moderate-activity RA and emphysema with no cardiac or renal disease and no pre-existing ILD being started on rituximab. A risk prediction scoring tool is being developed to be used in validation studies.

**Conclusion:**

This modified Delphi exercise defined and stratified the perceived risk factors for developing pneumotoxicity. Age, current smoking, high underlying rheumatological disease activity, HRCT definite UIP and honeycombing, severity and progression of pre-existing ILD were perceived to be the highest risk-factors.

**Supplementary Information:**

The online version contains supplementary material available at 10.1186/s12890-024-03287-0.

## Take home message

This modified Delphi exercise defined and stratified physician-perceived risk factors for developing pneumotoxicity in a field where the evidence-base is lacking.

## Introduction

Disease-modifying anti-rheumatic drugs (DMARDs) prescribed for chronic inflammatory rheumatic conditions are known to be associated with pneumotoxicity (drug-induced interstitial lung disease, DIILD). The potential risk factors that are associated with pneumotoxicity include the drug itself, the underlying rheumatological condition and other individual risk factors [1]. However estimating risk is difficult in an individualand this hampers provision of personalised risk/benefit counselling. Clinical drug trials are not designed to evaluate risk factors for pneumotoxicity and the majority of pneumotoxic events are reported via post-approval mechanisms [2, 3] or anecdotally. In the absence of empirical evidence, clinicians routinely employ their personal experiential knowledge of pneumotoxicity risk to counsel patients with rheumatic conditions. One way of exploring the parameters of risk is to collate this experiential knowledge. This lends itself to Delphi methodology [4] to address the research question ‘what are the risk factors for developing DIILD in patients with rheumatological conditions?’.

### Aims

The primary aim of the Delphi exercise was to ascertain physician-perceived risk factors associated with pneumotoxicity in patients with chronic inflammatory rheumatological conditions and stratify risks factors into low-, intermediate- and high-risk categories. The secondary aim was to explore differences between rheumatologist and pulmonologists’perceptions for ascertaining risk. An exploratory aim was to create a tool that could be used in future prospective studies of pneumotoxicity risk. The study did not address the potential pneumotoxicity risk associated with interaction of two or more drugs, the other risks of rheumatological drugs (mainly infection) or the benefits of DMARDs for treating the underlying rheumatological condition.

## Methods

International experts in rheumatology and pulmonology were identified by contact lists established for previous Delphi exercises, specialist societies and peer recommendations. Physicians were invited to respond to web-based e-Delphi questionnaires. No interviews were performed and physicians were anonymised such that respondents did not have sight of each other’s identity or individual responses.

The modified Delphi was structured as 3 tiers of questionnaires. Tier 1 sought to identify risk-factors for pneumotoxicity in patients with connective tissue disease (CTD). The parameters (patient factors and drug factors) and variables within each parameter were scoped by the steering committee based on their experience and knowledge of the literature and incorporated a wide spectrum of patient and drug factors that could plausibly be associated with risk of pneumotoxicity. The responses to questions such as ‘Do you consider smoking to be a risk factor for pneumotoxicity’ were restricted to ‘yes’, ‘no’ and ‘don’t know’. A pre-determinedthreshold of ≥70% agreement defined as ‘majority agreement’ was required for a risk variable to be progressed as a risk factor into Tier 2. The exception was for responses to the question: ‘Please identify which drugs you consider to be significantly associated with pneumotoxicity. Consider ‘significantly’ to be such that you would actively counsel the patient for a risk of pneumotoxicity’. For this question, a pre-defined threshold of > 30% affirmative responses led to the drug being progressed to Tier 2. All ‘don’t know’ responses were grouped with ‘no’. Respondents were provided with the grouped data results after Tier 1 and given the opportunity to changes their responses. Tier 2 employed a 6-point Likert scale and sought to stratify the risk parameters identified in Tier 1 into low (score 1 or 2), medium (score 3 or 4) and high risk (score 5 or 6) categories. The low-, medium- and high-risk categories were allocated from the median scores. Again, respondents were given the opportunity to modify their responses after they were given the Tier 2 grouped results. Tier 3 was a modification of a conventional Delphi exercise; six theoretical clinical scenarios were constructed based on the results of Tier 2; two scenarios were for cases that did not have pre-existing ILD, two for cases that had ILD but no data on progression of ILD prior to commencing drug and two for cases in which the nature of ILD disease progression was known by serial lung function data and computer tomography (CT) imaging.Respondents were asked to qualify the risk of pneumotoxicity for each scenario using a 5-point Likert scale (1 = very low, 2 = low, 3 = medium, 4 = high,5 = very high). All Tier questions are documented in the supplementary material.

Non-descriptive non-parametric statistical analysis was performed using Fischer’s exact test and Mann–Whitney U test.

### Ethics approval and consent to participate

Research Ethics approval was deemed unnecessary according to the Health Research Authority national guidelines (https://www.hra.nhs.uk/approvals-amendments/what-approvals-do-i-need/) since this was a study of professional practitioner opinion and did not involve patient participation. Informed consent was provided from all participants to take part in the study. The datasets analysed in this study are available from the corresponding author on reasonable request.

## Results

**Fig. 1:**
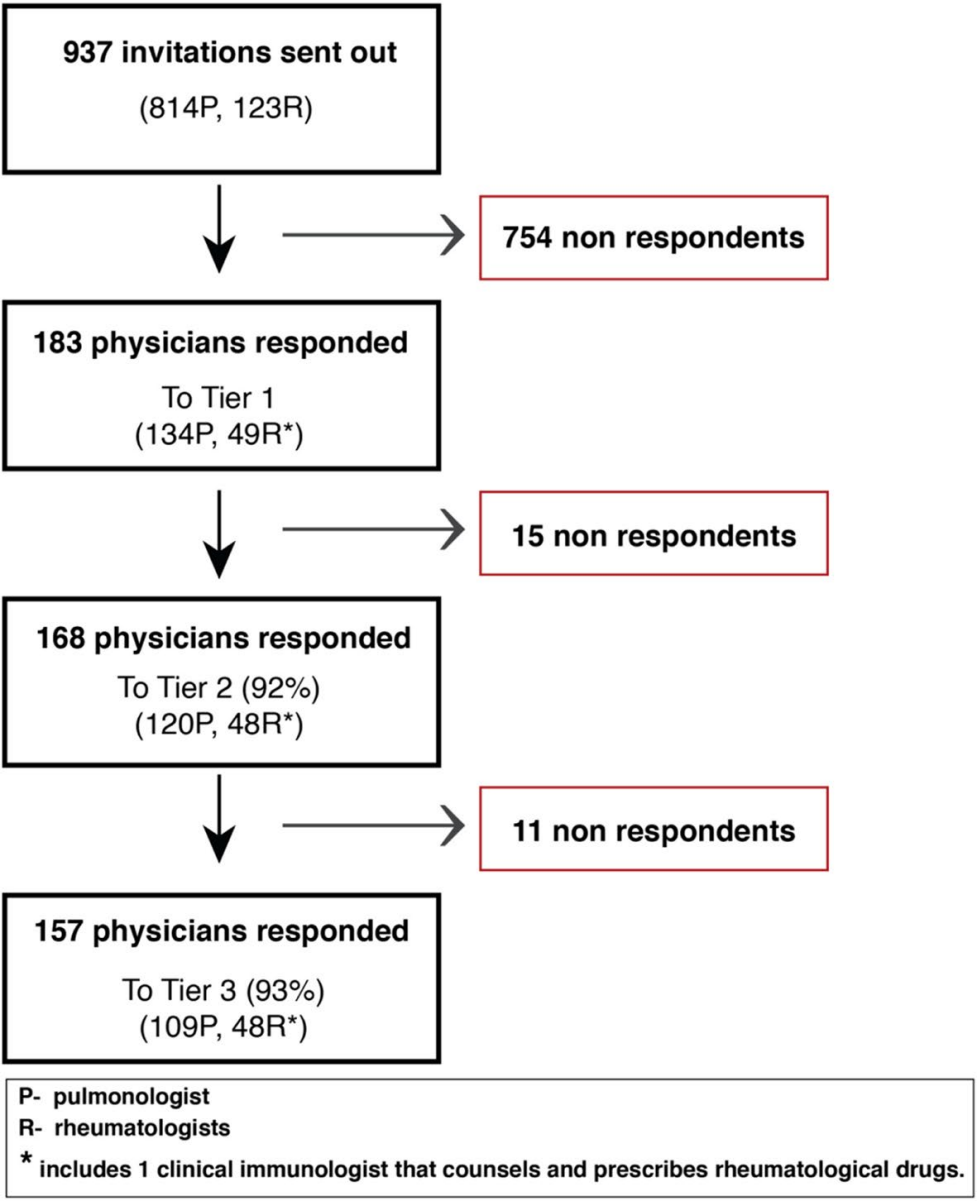
Consort diagram of respondents to each stage of the eDelphi process

183 physicians responded to Tier 1 (Fig. 1). Only 3.8% had less than 5 years’ experience in their specialty, and 82% had greater than 10 years’experience. 86% described themselves as working primarily in an academic/teaching/tertiary centre and 14% working in the community setting. In response to the question ‘How confident do you feel about assessing risk of pneumotoxicity in adults with DMARDS/Biologics and underlying rheumatological conditions?’, on a 7-point Likert scale where 0 = no confidence and 7 = extremely confident, the median response from both pulmonologists and rheumatologists was 5 indicating most respondents were moderately confident about assessing risk.

### Tier 1 results

Using the pre-defined cut-off of ≥ 70% of all respondents, age, smoking status, underlying rheumatological condition, activity of rheumatological condition, pre-existing ILD, static lung function, change in lung function over 1 year and progression of ILD over 1 year were thought to be significant risk factors for developing pneumotoxicity. There was no significant difference between pulmonologists’ and rheumatologists’ perceptions for these selected variables (Table 1). In contrast, over 70% of rheumatologists view COPD/emphysema and bronchiectasis as risk factors for DIILD, but significantly fewer pulmonologists do so(45% for COPD/emphysema and 51% for bronchiectasis). These variables did not meet the predefined threshold of ≥ 70% from all respondents and were therefore not taken forward to Tier 2. There were also significant differences between rheumatologists and pulmonologists in their perception of ethnicity, body mass index (BMI), and asthma as risk factors, but none of these variables reached the 70% threshold.

**Table 1:** Significance of patient variables when assessing risk of pneumotoxicity according to Rheumatologists and Pulmonologists. LVF: left ventricular failure, pulm HTN: pulmonary hypertension, CAD: coronary artery disease, ILD: interstitial lung disease, BMI: body mass index, COPD: chronic obstructive pulmonary disease. *P* values calculated using Fisher’s exact test

	**Combined** ***n*** **= 183 (%)**	**Rheumatologist** *n* **= 49 (%)**	**Pulmonologist** *n* **= 134 (%)**	***P*** **value**
Age	71	76	70	ns
Smoking	90	95	89	ns
Underlying rheumatological condition	89	93	87	ns
Activity of rheumatological condition	74	76	73	ns
Cardiac comorbidities: LVF, cor pulmonale, pulm HTN, significant CAD	76	86	75	ns
Renal function	74	73	74	ns
Pre-existing ILD	97	98	97	ns
Static lung function	93	91	94	ns
Progression of ILD (1 yr)	95	95	95	ns
Change in static lung function (1 yr)	90	86	92	ns
Previous episode of pneumotoxicity	75	70	77	ns
Gender	41	40	43	ns
Ethnicity	44	34	49	< 0.05
BMI	46	64	40	< 0.01
COPD and/or emphysema	55	79	45	< 0.001
Asthma	28	38	24	< 0.05
Bronchiectasis	57	71	51	< 0.01
Other respiratory comorbidities	33	30	33	ns
Diabetes Mellitus	26	14	31	< 0.05

**Fig. 2:**
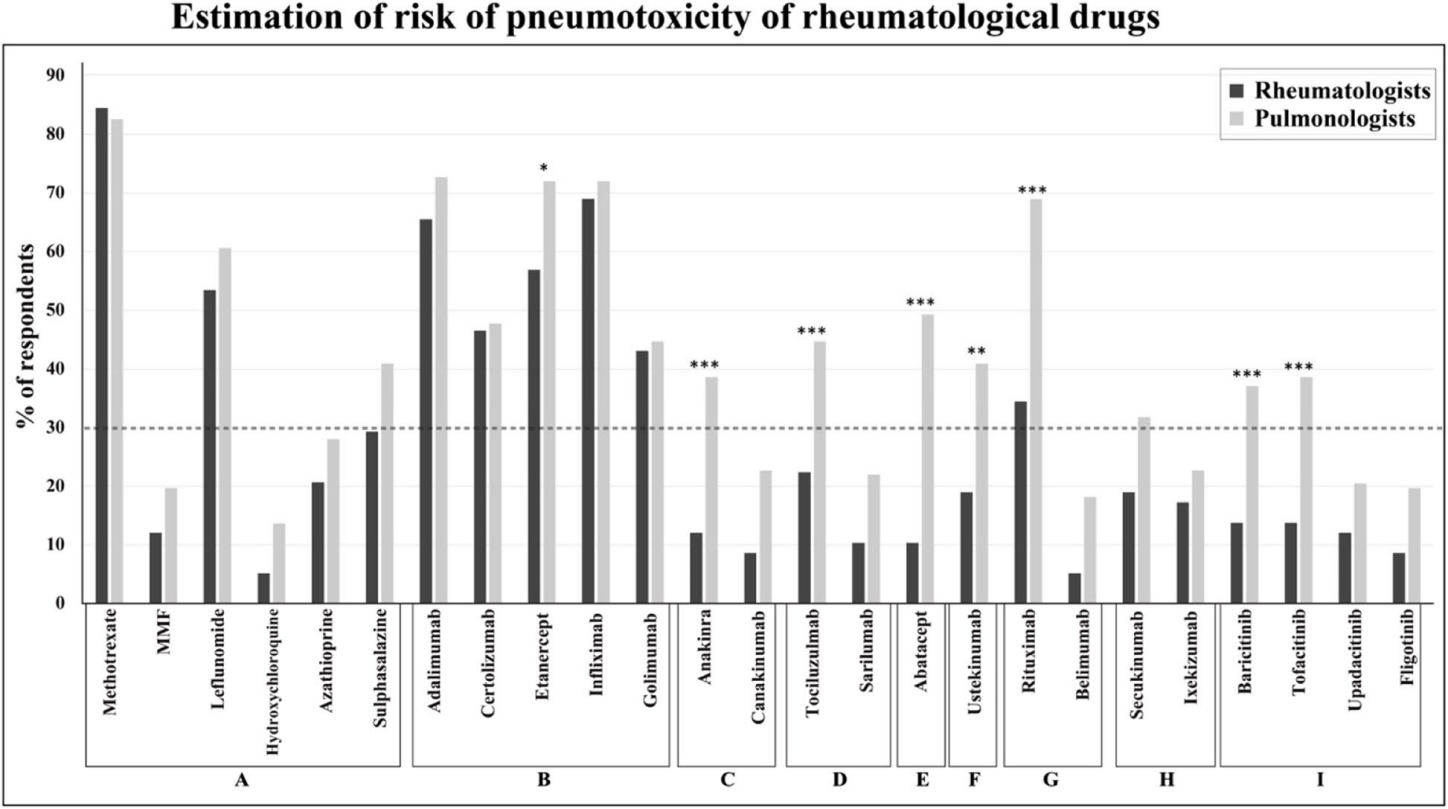
Risk of pneumotoxicity with commonly prescribed rheumatological drugs according to rheumatologists and pulmonologists. **A** = Disease modifying anti-rheumatic drugs, **B** = Anti-TNF, **C** = Anti-IL1, **D** = Anti-IL6, **E** = T-Cell modulator, **F** = Anti-IL12/23, **G** = B-Cell modulator, **H** = Anti-IL17, **I** = JAKSTAT inihibitors. *P* values calculated for those reaching the 30% threshold (dotted line): * < 0.05 ** < 0.01, *** < 0.001

Less than 30% of respondents indicated they would counsel patients for the risk of pneumotoxicity when starting azathioprine, belimumab, canakinumab, filgotinib, hydroxychloroquine, ixekizumab, mycophenolate mofetil (MMF), sarilumab, secukinumab and upadacitinib. These drugs were therefore not taken forward to Tier 2. Thirty percent or more of all respondents would counsel patients for pneumotoxicity when starting methotrexate, leflunomide, adalimumab, certolizumab, etanercept, infliximab, golimumab, anakinra, tocilizumab, abatacept, ustekinumab, rituximab, baricitinib and tofacitinib.Of these drugs, pulmonologists were significantly more likely than rheumatologists to perceive that etanercept, anakinra, tocilizumab, abatacept, ustekinumab, rituximab, baricitinib, and tofacitinib were associated with increased risk of pneumotoxicity (Fig. 2).

### Tier 2 results

DMARDs for which at least 30% of physicians would specifically counsel their patient about the potential for developing pneumotoxicity were assigned as low, medium and high risk according to median scores. Patient variables that 70% or more respondents designated as a significant risk factor when assessing pneumotoxicity were also assigned as low-, medium- and high-risk according to median score results. The data yielded from Tier 2 indicated that physicians perceived that pre-existing ILD and known progression of pre-existing ILD were important risk factors for developing pneumotoxicity. The results of all variables are presented in Table 2 (patient and drug variables), Table 3 (variables related to pre-existing ILD) and Table 4 (variables related to progression of pre-existing ILD). No drug was deemed as high risk whilst 10 drugs were deemed medium risk and 5 drugs were deemed low risk. High risk patient variables were age ≥ 70 years, current smoking, high rheumatological disease activity, definite UIP or honeycombing, ≥ 40% pre-existing ILD lung involvement on HRCT, TLCO < 30% predicted andat least an historical moderate worsening of ILD prior to initiating drug.

**Table 2:** Tier 2 results of low, medium and high-risk drug and patient variables for developing pneumotoxicity based on median values. RA: rheumatoid arthritis, SSc: systemic sclerosis, SLE: systemic lupus erythematosus, MCTD: mixed connective tissue disease

	**Low risk**	**Medium risk**	**High risk**
**Drug**	Sulphasalazine	Leflunomide	
	Tocilizumab	Adalimumab	
	Baricitinib	Certolizumab	
	Abatacept	Etanercept	
	Tofacitinib	Golimumab	
		Infliximab	
		Anakinra	
		Ustekinumab	
		Rituximab	
		Methotrexate	
**Age (years)**	< 49	50–69	≥ 70
**Smoking**	never	ex	current
**Connective tissue disease**	Psoriatic arthropathy, Ankylosing spondylitis	RA, SSc, SLE, Primary Sjogren's, MCTD, Anti-synthetase syndromes, systemic vasculitis, sarcoidosis	
**Connective tissue disease activity**	Low	Medium	High
**Cardiac comorbidities**	Nil or presence of coronary artery disease	Left ventricular failure Cor pulmonale, Pulmonary hypertension	
**Renal comorbidities: estimated glomerular filtration rate**	≥ 60	< 60	
**Previous pneumotoxicity**	NO	YES

**Table 3:** Tier 2 results of low, medium and high-risk variables in the presence of pre-existing interstitial lung disease. ILD: Interstitial lung disease, UIP: usual interstitial pneumonia, FVC: Forced vital capacity, TLCO: Transfer factor for carbon monoxide, CT: computer tomography

Pre-existing ILD	Low risk	Medium risk	High risk
ILD pattern on CT		Probable UIP, fibrosis with indeterminate pattern, fibrosis with other pattern, inflammation no fibrosis	Definite UIP, Honeycombing
Volume of lung affected by ILD on CT (%)	< 20	20–39	≥ 40
Lung function: FVC, % predicted	≥ 60	< 60	
Lung function: TLCO, % predicted	≥ 80	30–79	< 30

**Table 4:** Tier 2 results of low, medium and high-risk variables in the presence of serial computer tomography (CT) and lung function data in pre-existing interstitial lung disease (ILD). HRCT: high resolution CT, FVC: Forced vital capacity

Serial ILD data available	Low risk	Medium risk	High risk
Change in HRCT	No / minimal	mild	Moderate or greater
Change in lung function: FVC, % predicted	< 5%	≥ 5%	

### Tier 3 results

The clinical scenarios presented to the respondents in Tier 3 were cases that incorporated the lowest risk variables and highest risk variables that could be associated with patients that had no pre-existing ILD (scenario 1 and 2), patients with pre-existing ILD but no serial information (scenario 3 and 4) and patients with pre-existing ILD and serial information that informed progression (scenario 5 and 6) respectively. The results are presented in Table 5. Scenarios that only included low risk drug and patient variables (scenario 1, 3 and 5) were deemed as low or very low risk. Scenarios that only included the highest risk categories for patient and drug variable (scenarios 2, 4 and 6) were deemed medium or high risk. For example, a 70-year-old current smoker with high-activity rheumatoid arthritis (all high-risk variables) but no pre-existing ILD, was deemed medium risk if being started on methotrexate. This was the maximum risk for any scenario that does not have pre-existing ILD. There was no scenario, including scenario 6 wherein every maximum risk category was applied, that resulted in a ‘very high risk’ outcome.

**Table 5:** Characteristics of 6 scenarios presented in Tier 3

Scenario no	Characteristics^a^	Qualitative risk
**No ILD**		
1	49yrs Ankylosing spondylitis Abatacept	Very low
2	70yrs Rheumatoid arthritis Methotrexate	Medium
**With ILD**		
3	Scenario 1 AND Indeterminate fibrosis < 20% volume on CT FVC > 60% TLCO > 80%	Low
4	Scenario 2 AND UIP > 40% volume on CT FVC < 60% TLCO < 30%	High
**With ILD and serial data**		
5	Scenario 3 AND No/Minimal progression on CT < 5% decline FVC < 10% decline TLCO	Low
6	Scenario 4 AND Moderate progression on CT > 5% decline FVC > 10% decline TLCO	High

^a^The full scenariosare described in the supplementary material

## Discussion

DIILD is well recognised and is an occasionally fatal consequence of rheumatological drugs. One systematic review estimated that the prevalence of DIILD with methotrexate, leflunomide and tumour necrosis factor inhibitors (TNFi) in rheumatoid arthritis patients is around 1% [1]. The evidence base for risk factors for DIILD is also sparse and is based on clinical trial, pharmacovigilance and registry databases, and case-reports. Systematic reviews [5, 6] and a meta-analysis [7]of DIILD including drugs used in rheumatic diseases do not specifically address risk-factors for pneumotoxicity and conclude that the majority of relevant studies are of low quality. Recent expert guidelines and a comprehensive review either do not comment on risk-factors [8] or variably cite pre-existing ILD, older age, previous use of DMARDs and diabetes as risk-factors for methotrexate-induced ILD [9–11]. However, the comprising studies for these data are small with 48 cases in approximately 1350 exposed patients [12–14]. Therefore the evidence for risk factors for DIILD is poor.

The aim of this global Delphi exercise was to ascertain physician-perceived risk-factors for pneumotoxicity. There are no equivalent Delphi studies to which these data can be readily compared. Fewer than 30% of respondents counsel patients for pneumotoxicity when starting azathioprine, belimumab, canakinumab, filgotinib, hydroxychloroquine, ixekizumab, mycophenolate mofetil, sarilumab, secukinumab or upadacitinib. These drugs were deemed ‘safe’ in terms of pneumotoxicity by the majority of physicians and indeed there are no or very few citations of DIILD in the Pneumotox database (www.pneumotox.com) for these drugs. Pulmonologists generally perceived a higher risk of pneumotoxicity with drugs compared to rheumatologists. For example, significantly more pulmonologists than rheumatologists would counsel patients for pneumotoxicity starting abatacept, anakinra, baricitinib, tofacitinib, rituximab, tocilizumab, or ustekinumab. Importantly, pulmonologists represented around 70% of total respondents and are likely to see the majority of cases of significant pneumotoxicity, but they are not the primary prescriber of these drugs. This likely explains why these drugs are preceived to be as more risky by pulmonologists than rheumatologists. Methotrexate is the most well reported and recognised cause of DMARD-associated DIILD, but there is growing appreciation that this drug might delay the onset of rheumatoid-arthritis associated ILD [15, 16].

The majority (≥ 70%) of physicians believed that history of previous DMARD associated pneumotoxicity, age, smoking, nature and activity of underlying CTD, renal function, left ventricular failure, pulmonary hypertension/cor-pulmonale and presence, severity and previous progression of pre-existing ILD are relevant risk factors despite the poor evidence-base for these perceptions. Although diabetes is cited as a risk factor in the literature based on relatively small studies, only 26% respondents felt this was the case.

In particular more than 90% of pulmonologists, rheumatologists or both perceived that the presence of ILD, ILD severity, ILD progression prior to starting DMARDs and smoking are risk factors for developing pneumotoxicity. Usual Interstitial Pneumonia (UIP) pattern or honeycombing on HRCT scan was deemed to be a higher risk factor than other patterns of ILD. The Delphi exercise did not seek to address the underlying reasoning for physician-perceived risk factors but the majority felt confident in counselling patients of the risks (median score 5 where 0 is ‘no confidence and 7 is ‘extremely confident). The assumption is that physicians use their experiential knowledge, and inherent biases, to estimate risk. The vast majority of responding physicians perceive that underlying progressive severe ILD and current smoking are high risk factors, so it may be speculated that physicians perceive that introducing a drug that perturbs immune function into an environment that already harbours inflammation or fibrosis may trigger DIILD. It is also pertinent that several of the perceived risk factors for DIILD, such as age, forced vital capacity (FVC), Transfer factor for carbon monoxide (TLCO), UIP pattern on HRCT and historical progression of ILD, are also recognised risk factors for future progression of fibrotic ILDs [17]. Awareness of this may influence respondents’ perception of risk factors for DIILD.

There are some strengths to this study. The evidence base for risk factors is poor which often drives variable decision-making but the clinical problem is significant and therefore appropriate for a Delphi exercise. The global experience of the respondents is substantial and the number of respondents is reasonable with < 15% attrition between Tiers, but this is still a relatively small sample.

There are a number of limitations to this study. Whilst potential participants were contacted in all continents and more than 40 countries, most respondents were from Europe and North America and will inevitably reflect their local practice and perceptions. For example, ethnicity was not thought to be a risk factor for DIILD by the majority of respondents. However, it is likely that genetic predetermination and ethnicity are risk factors for pneumotoxicity, as suggested in acute exacerbation of ILD and DIILD due to the tyrosine kinase inhibitors, methotrexate and leflunomide in South East Asia [18, 19]. The study is best described as a modified-Delphi exercise; whilst respondents were given feedback and opportunity to revise answers, complete consensus was not required. Rather a predetermined majority of ≥ 70% was deemed sufficient. Finally, none of the benefits of these potentially transformative drugs are captured in this study. In clinical practice, physicians would convey both risks and benefits when counselling patients.

A risk-evaluation tool derived from the tables will be developed and its performance evaluated in a prospective study. A validated risk evaluation tool could be used as an adjunct to decision making particularly with regard to treatment choice. For example, patients deemed at very low or low risk may only require lung function or radiologicalfollow up if respiratory symptoms arise whilst patients deemed at high-risk would require careful counselling about the development of lung symptoms and regular monitoring for early signs of respiratory compromise after starting DMARD.

## Conclusions

Counselling patients for developing pneumotoxicity with rheumatological drugs is common in clinical practice but the evidence base for the associated risk factors is poor. This modified international Delphi exercise identified physician-perceived risk factors for developing pneumotoxicity and forms the basis of a risk evaluation tool that if validated may aid clinical decisions in the absence of suitable evidence.

## Supplementary Information

Supplementary Material 1.

Supplementary Material 2.

Supplementary Material 3.

## Data Availability

The datasets analysed in this study are available from the corresponding author on reasonable request.
