# Supplementary material for: A modified Delphi exercise in physician-perceived risk factors for drug-induced pneumotoxicity in patients with rheumatological disease
Source: BMC Pulm Med. 2024 Oct 31;24:547. doi: 10.1186/s12890-024-03287-0 (PMC11529426; doi:10.1186/s12890-024-03287-0)
Supplement: Supplementary file 1 — Supplementary Material 1. [file 12890_2024_3287_MOESM1_ESM.pdf]

Pneumotoxicity Associated With Rheumatological Drugs (PASSWRD) Tier 1

An e-Delphi exercise to reach consensus on risk of drug-induced pneumotoxicity in patients with rheumatological disease

Section 1

Tier 1

About you. All responses are anonymous.

1. **Please enter the email address that this invitation was sent to:** (for subsequent engagements if you would prefer to use a different email please contact loth.delphipneumotoxicity@nhslothian.scot.nhs.uk)

Enter your answer

2. **What is your primary specialty?**

Rheumatology☐ Pulmonology☐

3.**Years of experience in your specialty (in years)**

1-4 ☐ 5-9 ☐ 10-20 ☐ > 20 ☐

4.**How confident do you feel about assessing risk of pneumotoxicity in adults with DMARDS/Biologics and underlying rheumatological conditions?**

1= no confidence. 7= extremely confident.

1☐ 2☐ 3☐ 4☐ 5☐ 6☐ 7☐

5. **PATIENT FACTOR DOMAINS**

We would like to compile a list of relevant patient factors for Tier 2 of this Delphi exercise.

Which of the following patient factors do you consider when assessing risk of developing pneumotoxicity?

Age

Yes☐ No☐ Don't know ☐

6. **Gender**

Yes☐ No☐ Don't know☐

7.**Ethnicity**

Yes ☐ No ☐ Don't know ☐

8. **Smoking**

Yes ☐ No ☐ Don't know ☐

9. **BMI**

Yes ☐ No ☐ Don't know ☐

10. **Underlying rheumatological diagnosis**

Yes ☐ No ☐ Don't know ☐

11. **Activity of underlying rheumatic disease**

Yes ☐ No ☐ Don't know ☐

12.**If answered "no" or "I don't know" to Q11, please go to Q13. If answered "yes" please specify which measure of activity you consider when assessing risk of pneumotoxicity (tick all that apply)**

Disease activity score (e.g. DAS28, BASDAI, PsARC, SLEDAI or other) ☐

Imaging score (MRI, US etc) ☐ ESR ☐ CRP ☐ Ferritin ☐

Complement ☐ Autoimmune serological titre (RhF, CCP, Jo-1 etc) ☐ Serum ACE ☐

13. **Pre-existing interstitial lung disease (ILD)**

Yes ☐ No ☐ Don't know ☐

14. **If you responded "no" or "I don't know" to Q13, please go to Q20. If you responded "yes" , please indicate which features on HRCT you consider when assessing risk of pneumotoxicity**

Pattern of disease (for example UIP, NSIP, etc.)

Yes ☐ No ☐ Don't know ☐

15. **Presence of fibrosis (honeycombing, traction bronchiectasis)**

Yes ☐ No ☐ Don't know ☐

16. **Presence of 'inflammation' (ground glass or air-space opacification)**

Yes ☐ No ☐ Don't know ☐

17. **Presence of lung nodules**

Yes ☐ No ☐ Don't know ☐

18. **Volume of lung affected by ILD on HRCT**

Yes ☐ No ☐ Don't know ☐

19. **Progression of ILD between serial HRCT scans**

Yes ☐ No ☐ Don't know ☐

20. **COPD and/or emphysema**

Yes ☐ No ☐ Don't know ☐

21. **Asthma**

Yes ☐ No ☐ Don't know ☐

22. **Bronchiectasis**

Yes ☐ No ☐ Don't know ☐

23.**Other respiratory co-morbidities - please specify**

Enter your answer

24.**Static Lung function measurements (spirometry, gas transfer)**

Yes ☐ No ☐ Don't know ☐

25.**If you responded "no" or "don’t know" to Q24, please go to Q30. If you responded "yes", please indicate which features on static lung function would you consider when assessing risk of pneumotoxicity**
FEV1

Yes ☐ No ☐ Don't know ☐

26. **FVC**

Yes ☐ No ☐ Don't know ☐

27.**FEV1/FVC ratio**

Yes ☐ No ☐ Don't know ☐

28.**TLCO**

Yes ☐ No ☐ Don't know ☐

29.**KCO (=TLCO corrected for alveolar volume)**

Yes ☐ No ☐ Don't know ☐

30.**Change in static lung function between historical serial measurements (if available)**

Yes ☐ No ☐ Don't know ☐

31.**If answered "no" or "don't know" to Q30, please go to Q35. If answered "yes" , please indicate which features of serial static lung function measurement (if available) do you consider when assessing risk of pneumotoxicity.**

Change in FEV1

Yes ☐ No ☐ Don't know ☐

32. **Change in FVC**

Yes ☐ No ☐ Don't know ☐

33. **Change in TLCO**

Yes ☐ No ☐ Don't know ☐

34. **Change in KCO**

Yes ☐ No ☐ I don't know ☐

35. **Desaturation on formal exercise testing (e.g. 6MWT, shuttle etc)**

Yes ☐ No ☐ Don't know ☐

36. **Walking distance on formal exercise testing (e.g. 6MWT, shuttle etc)**

Yes ☐ No ☐ Don't know ☐

37. **Please indicate which non-respiratory co-morbidities you consider when assessing the risk of pneumotoxicity (tick all that apply)**

Cardiac ☐ **Liver** ☐ **GI tract** ☐ **Renal** ☐
Neurological ☐ **Diabetes mellitus** ☐ **Metabolic/endocrine** ☐
Haematological ☐ **Psychiatric** ☐ **None of the above** ☐

38. **DRUG FACTOR DOMAINS**

We would like to compile a list of relevant rheumatological drugs for Tier 2 of this Delphi exercise.

Please identify which drugs you consider to be significantly associated with pneumotoxicity. Consider 'significantly' to be such that you would actively counsel the patient that there is a risk of pneumotoxicity.
Conventional DMARDs (tick all that apply)

Azathioprine ☐ Methotrexate ☐ Leflunomide ☐ Sulfasalazine ☐

Hydroxychloroquine ☐ MMF ☐ None of the above ☐

39. **Anti-TNF (tick all that apply)**

Adalimumab ☐ Certolizumab ☐ Etanercept ☐

Golimumab ☐ Infliximab ☐ None of the above ☐

40. **Do you consider that individual drugs within the anti-TNF class have different risks of pneumotoxicity?**

Yes ☐ No ☐ Don't know ☐

41. **Anti-IL1 (tick all that apply)**

Anakinra ☐ Canakinumab ☐ None of the above ☐

42. **Do you consider that individual drugs within the anti-IL1 class have different risks of pneumotoxicity?**

Yes ☐ No ☐ Don't know ☐

43. **Anti-IL6, (tick all that apply)**

Sarilumab ☐ Tocilizumab ☐ None of the above ☐

44. **Do you consider that individual drugs within the anti-IL6 class have different risks of pneumotoxicity?**

Yes ☐ No ☐ Don’t know or not applicable ☐

45.**Anti IL12/23**

Ustekinumab ☐ None of the above ☐

46. **Do you consider that individual drugs within the anti-IL12/23 class have different risks of pneumotoxicity?**

Yes ☐ No ☐ Don’t know or not applicable ☐

47. **Anti-IL17 (tick all that apply)**

Ixekizumab ☐ Secukinumab ☐ None of the above ☐

48. **Do you consider that individual drugs within the anti-IL17 class have different risks of pneumotoxicity?**

Yes ☐ No ☐ Don’t know or not applicable ☐

49. **JAK-STAT inhibitors (tick all that apply)**

Baricitinib ☐ Tofacitinib ☐ Upadacitinib ☐

Filgotininb ☐ None of the above ☐

50. **Do you consider that individual drugs within the JAK-STAT inhibitor class have different risks of pneumotoxicity?**

Yes ☐ No ☐ Don’t know or not applicable ☐

51. **T-cell modulator**

Abatacept ☐ None of the above ☐

52. **B-cell depleter or modulator (tick all that apply)**

Belimumab ☐ Rituximab ☐ None of the above ☐

53. **Do you consider that individual drugs within the B-cell deplete/modulator class have different risks of pneumotoxicity?**

Yes ☐ No ☐ Don't know or not applicable ☐

54. **Other drugs. In the free text box please indicate any other licensed drugs that you currently use in CTD and you associate with pneumotoxicity**
