# Supplementary material for: A modified Delphi exercise in physician-perceived risk factors for drug-induced pneumotoxicity in patients with rheumatological disease
Source: BMC Pulm Med. 2024 Oct 31;24:547. doi: 10.1186/s12890-024-03287-0 (PMC11529426; doi:10.1186/s12890-024-03287-0)
Supplement: Supplementary file 2 — Supplementary Material 2. [file 12890_2024_3287_MOESM2_ESM.pdf]

Pneumotoxicity Associated With Rheumatological Drugs (PASSWRD) Tier 2

Patient factor domains

What level of risk do you feel applies to the following domains?

Note **1=lowest risk, 6= highest risk**. Please provide the risk level relative to the risk of other items in the **same** domain, **and** to the risk of items in **other** domains.

For example, if you feel increasing age is an important independent risk factor, enter a low score in 'lower age' and a higher score for 'high age'. If you think increased age is a greater risk factor than never smoked, enter a higher score for increased age than for never smoked. Please use the whole scale such that if you think e.g. the presence of RA confers the highest independent risk amongst all the patient factor items, select '6' for this item.

1.**Please enter your email address that this invitation was sent to:**

Enter your answer

2.Please indicate the level of risk of developing pneumotoxicity with respect to

Age: (1=lowest risk, 6=highest risk) Required to answer. Liker.

	1	2	3	4	5	6
**Age <40yrs**						
**Age 40-49yrs**						
**Age 50-59yrs**						
**Age 60-69yrs**						
**Age 70-79yrs**						
**Age >80yrs**						

3.Please indicate the level of risk of developing pneumotoxicity with respect to **Smoking: (1=lowest risk, 6=highest risk)**

	1	2	3	4	5	6
**Never smoked**						
**Ex-smoker**						
**Current smoker**						

4.Please indicate the level of risk of developing pneumotoxicity with respect to **Underlying CTD: (1=lowest risk, 6=highest risk)**

	1	2	3	4	5	6
**Rheumatoid Arthritis**						
**Systemic sclerosis**						
**Systemic Lupus Erythematosus**						
**Primary Sjogren's**						
**Mixed Connective Tissue Disease**						
**Antisynthetase syndromes**						
**Psoriatic arthritis**						
**Ankylosing spondylitis**						
**Systemic Vasculitis**						
**Sarcoidosis**						

5. Please indicate the level of risk of developing pneumotoxicity with respect to **Activity of underlying CTD** based on **disease activity score, ESR, CRP and autoimmune serological titre**: **(1=lowest risk, 6=highest risk)**

	1	2	3	4	5	6
**Low disease activity**						
**Medium disease acitivity**						
**High disease activity**						

6.With respect to the risk of **Interstitial lung disease (ILD) on HRCT,** please indicate which level of risk is associated with the presence of the following features:

Please provide the risk level relative to the risk of other items in the same domain, and to the risk of items in other patient factor domains. (1=lowest risk, 6=highest risk). For example if you feel presence of UIP is a greater risk factor than the presence of Inflammation, enter a higher score for UIP than Inflammation. If you think that the presence of NSIP is a greater risk factor than CTD disease activity, enter a higher score for NSIP than disease activity.

	1	2	3	4	5	6
**No ILD**						
**A definite usual interstitial pneumonia (UIP) pattern**						

	1	2	3	4	5	6
**A probable UIP pattern**						
**Fibrosis with indeterminate pattern**						
**Fibrosis with other patterns e.g. NSIP/ chronic hypersensitivity pattern / Sarcoidosis**						
**Presence of honeycombing on HRCT**						
**Presence of Inflammation (reported as ground glass, air-space opacification) without fibrosis**						

7.Please indicate the level of risk of developing pneumotoxicity with respect to **Volume of total ILD on HRCT: (1=lowest risk, 6=highest risk)**

	1	2	3	4	5	6
**<10%**						
**10-19%**						
**20-29%**						
**30-39%**						
**40-49%**						
**>50%**						

8.Please indicate the level of risk of developing pneumotoxicity with respect to **Progression of ILD on serial HRCT if available: (1=lowest risk, 6=highest risk)**

	1	2	3	4	5	6
**No or minimal progression within 1 year**						
**Mild progression within 1 year**						
**Moderate or greater progression within 1 year**						

9.With respect to the risk of **Static lung function measurements (spirometry, gas transfer),** please indicate which level of risk is associated with the presence of the following features:

Please provide the risk level relative to the risk of other items in the same domain, and to the risk of items in other patient factor domains. 1=lowest risk, 6=highest risk. For example if you feel presence of TLCO <30% of predicted is a greater risk factor than FVC >80% predicted enter a

higher score for the former. If you think that TLCO <30% of predicted is a greater risk factor than presence of UIP on HRCT, enter a higher score for the former.

	1	2	3	4	5	6
**FVC >80% predicted**						
**FVC 60-80% predicted**						
**FVC 45-60% predicted**						
**FVC <45% predicted**						
**TLCO >80% predicted**						
**TLCO 50-80% predicted**						
**TLCO 30-50% predicted**						
**TLCO <30% predicted**						
**KCO >80% predicted**						
**KCO 50-80% predicted**						
**KCO 30-50% predicted**						
**KCO <30% predicted**						

10.With respect to the risk of **Change in serial static lung function measurements (spirometry, gas transfer),** please indicate which level of risk is associated with the presence of the following features:

Please provide the risk level relative to the risk of other items in the same domain, and to the risk of items in other patient factor domains. 1=lowest risk, 6=highest risk. For example if you feel decline in TLCO is of predicted is a greater risk factor than the decline in FVC enter a higher score for the former. If you think that decline in TLCO is a greater risk factor than presence of UIP on HRCT, enter a higher score for the former.

	1	2	3	4	5	6
**Decline in FVC of >10% within 1yr**						
**Decline in FVC of 5-10% within 1yr**						
**Decline in FVC<5% within 1 year**						
**Decline in TLCO of >10% within 1yr**						
**Decline in TLCO of <10% within 1yr**						

11.When considering **Desaturation on exercise testing** which of the following do you consider as a risk of developing pneumotoxicity?

	1	2	3	4	5	6
**No desaturation**						
**Desaturation to <92% and >88%**						
**Desaturation to </=88%**						

12.Please indicate the level of risk of developing pneumotoxicity with respect to **Cardiac co-morbidities: (1= lowest risk, 6= highest risk)**

	1	2	3	4	5	6
**Left ventricular failure**						
**Cor pulmonale**						
**Pulmonary hypertension**						
**Significant coronary artery disease**						

13.Please indicate the level of risk of developing pneumotoxicity with respect to **Renal co-morbidities, measured as estimated glomerular filtration rate (EGFR): (1= lowest risk, 6= highest risk)**

	1	2	3	4	5	6
**EGFR <30%**						
**EGFR 30 - 60**						
**EGFR >60%**						

14.**Medication/drug factors**

The following are the drugs that, when started, at least 30% of respondents would counsel their patients for the risk of pneumotoxicity. Please provide the risk level of **each drug relative to the risk of other drugs** on this list, **but <u>not</u> relative to the risk of other patient factor domains**. **1=lowest risk, 6= highest risk.**

For example if you feel methotrexate has the highest risk relative to the other drugs, please enter 6 for methotrexate. If you think abatacept has the lowest risk relative to the other drugs, then please enter 1. Please use the whole scale such that the drug which you believe causes the highest risk **scores 6** and the drug with the lowest risk **scores 1**.

	1	2	3	4	5	6	Don't know
**Sulphasalazine**							
**Leflunomide**							
**Methotrexate**							
**Adalimumab (Humira, Hyrimozetc)**							
**Certolizumab (Cimzia)**							
**Etanercept (Enbrel, Erelzietc)**							
**Golimumab (Simponi, Simponi Aria)**							
**Infliximab (Remicare, Renflexisetc)**							
**Anakinra (Kineret)**							
**Tocilizumab**							
**Ustekinumab**							
**Tofacitinib**							
**Baricitinib**							
**Abatacept (Orencia)**							
**Rituximab (Rituxan)**							

15.Please indicate what level of risk you would assign to **prescribing a DMARD/Biologic that is associated with a risk of pneumotoxicity** if a previously tried DMARD/biologic (regardless of class of drug) had to be stopped due to pneumotoxicity i.e. past medical history of drug discontinuation due to pneumotoxicity? **1=lowest risk, 6=highest risk**

Level of risk of prescribing a new **DMARD/biologic** in patients with **past history of pneumotoxicity**

1 2 3 4 5 6 Don't know
