# Supplementary material for: A modified Delphi exercise in physician-perceived risk factors for drug-induced pneumotoxicity in patients with rheumatological disease
Source: BMC Pulm Med. 2024 Oct 31;24:547. doi: 10.1186/s12890-024-03287-0 (PMC11529426; doi:10.1186/s12890-024-03287-0)
Supplement: Supplementary file 3 — Supplementary Material 3. [file 12890_2024_3287_MOESM3_ESM.pdf]

Pneumotoxicity Associated With Rheumatological Drugs (PASSWRD)

Tier 3

Please read carefully: Your responses for Tier 2 enabled us to assign all relevant clinical variables into low, medium and high risk categories. **Please refer to Tables 1 and 2 in the Tier 2 feedback**. We now present 6 clinical scenarios. The scenarios are designed to contain all the relevant risk factor information and cover the whole spectrum of risks based on your responses.

For each scenario we would like you to **assign the level of risk you would convey to the patient for developing pneumotoxicity**. Please choose ONE of the categories from 'Very low', 'Low', 'Medium', 'High' or 'Very high'.

We will then ask you to **approximately quantify the risk** for each category. This is an important patient-centered question and one of the aims of this study and we would encourage you to answer with an estimated risk. However it may be difficult even for experts to quantify risk, hence there is a 'don't know' option.

At the end of the questionnaire, for demographic purposes please enter **YOUR** country of practice/residence and details about your institution.

1. **Please enter your email address that this invitation was sent to:**

Enter your answer

2. A patient aged less than 49yrs with ankylosing spondylitis is being considered for treatment with abatacept.

The patient has no ILD. They have low disease activity score, are a non-smoker, no significant cardiac or renal disease and have never experienced pneumotoxicity with any other DMARD therapy before.

How would you convey to the patient the risk of developing neurotoxicity? Please choose from ONE of the options listed below:

Very Low ☐ Low ☐ Medium ☐ High ☐ Very high ☐

3.**The same patient with ankylosing spondylitis is being considered for treatment with abatacept but the patient has ILD of indeterminate pattern, with fibrosis but not honeycombing. The volume of disease on CT is <20% and the lung function shows FVC > 60% predicted and TLCO >80% predicted.**

How would you convey to the patient the risk of developing pneumotoxicity? Please choose from ONE of the options listed below:

Very Low ☐ Low ☐ Medium ☐ High ☐ Very high ☐

4.The same patient with ankylosing spondylitis and ILD of indeterminate pattern is being considered for treatment with abatacept and the patient has serial HRCT and lung function tests. The HRCT shows no or minimal progression over <1 year. The lung function shows less than 5% decline in FVC and less than 10% decline in TLCO.
How would you convey to the patient the risk of developing pneumotoxicity? Please choose from ONE of the options listed below:

Very Low☐ Low ☐ Medium☐ High☐ Very high☐

5. **A patient aged over 70yrs with rheumatoid arthritis is being considered for treatment with methotrexate. The patient has no ILD. They have high disease activity, are a current smoker, and have pulmonary hypertension, renal failure with EGFR <60 and has experienced pneumotoxicity with a previous.**

Very Low☐ Low ☐ Medium ☐ High ☐ Very high☐

6. **The same patient with rheumatoid arthritis is being considered for treatment with methotrexate and the patient and ILD of UIP pattern. The volume of disease on the scan is >40%. Recent lung function shows FVC <60% predicted and TLCO <30% predicted.** How would you convey to the patient the risk of developing pneumotoxicity? Please choose from ONE of the options listed below:

Very Low ☐ Low ☐ Medium ☐ High ☐ Very high☐

7. **The same patient with rheumatoid arthritis and ILD of UIP pattern is being considered for treatment with methotrexate and the patient has serial HRCT and lung function data. The HRCT shows at least moderate progression over <1yr. The lung function shows more than a 5% decline in FVC and more than 10% in TLCO. How would you convey to the patient the risk of developing pneumotoxicity?** Please choose from ONE of the options listed below:

Very Low ☐ Low ☐ Medium ☐ High ☐ Very high ☐

8. If the patient asks you to **quantify** their approximate risk how would you respond? Please enter answers in the form '**1 in X'.** So if you think 'very low risk' means approximately 1 in 1000, please enter ' 1 in 1000' into the relevant box. If you think 'very high risk' means approximately 1 in 2, please enter '1 in 2' in the relevant box. If you would counsel the patient that you are not able to quantify the risk, please enter 'don't know'.
Very low:
Enter your answer

9. **Low**:
Enter your answer

10. **Medium**:

Enter your answer

11. **High**:

Enter your answer

12. **Very high**:

Enter your answer

13. Which country are **YOU** based in? If your country of residence is different to the country that you practice in, please enter the country you practice in.

Enter your answer

14. Please indicate where **YOU** primarily care for patients. If you work for multiple institutions, please enter the institution that you spend the majority of your time with.

Academic and/or tertiary referral centre

Community hospital/unit
